# The role of curcumin in modulating nutritional status and susceptibility to *Mycoplasma* pneumoniae infection in children

**DOI:** 10.3389/fphar.2025.1651875

**Published:** 2025-09-11

**Authors:** Chun-Jing Liu, Wei Liu, Hong-Xiu Yang, Li-Hua Li

**Affiliations:** Department of Pediatrics, Beijing Luhe Hospital, Capital Medical University, Beijing, China

**Keywords:** antibiotic therapy, curcumin, immunomodulatory effects, inflammatorycytokines, *Mycoplasma* pneumoniae

## Abstract

**Background:**

*Mycoplasma pneumoniae* remains a leading cause of pediatric respiratory infections, often resulting in prolonged symptoms, hospitalization, and systemic inflammation. Curcumin has been proposed as an adjunctive therapy due to its anti-inflammatory and immunomodulatory properties. This study retrospectively evaluated the association between adjunctive curcumin supplementation and clinical, immunological, and nutritional outcomes in children with confirmed *M. pneumoniae* infection.

**Methods:**

We performed a retrospective observational study of children aged 1–12 years at Beijing Luhe Hospital (September 2023–May 2024). Based on charted treatment, 160 patients were categorized into a curcumin-supplemented group (n = 80; standardized 95% curcuminoids, 20 mg/kg/day, with antibiotics) or a control group (n = 80; antibiotics alone). Outcomes included duration of fever/cough, hospitalization, severe complications, inflammatory markers—C-reactive protein (CRP), interleukin-6 (IL-6), tumor necrosis factor-alpha (TNF-α)—pathogen-specific antibodies, and nutritional indices (body mass index [BMI], hemoglobin, serum albumin). Adverse events (AEs) were summarized.

**Results:**

Baseline characteristics were comparable between groups (all p > 0.05). The curcumin group had shorter fever (3.2 ± 1.1 vs. 4.5 ± 1.3 days, p = 0.01) and cough durations (5.4 ± 2.0 vs. 7.1 ± 2.5 days, p = 0.02), lower hospitalization rates (1.25% vs. 10.0%, p = 0.02), and fewer severe complications (2.5% vs. 12.5%, p = 0.03). Greater reductions were observed in CRP (−9.6 ± 5.1 vs. −1.8 ± 4.7 mg/L, p = 0.011), IL-6 (−15.1 ± 6.3 vs. −2.5 ± 5.8 pg/mL, p = 0.01), and TNF-α (−9.6 ± 5.4 vs. −1.7 ± 5.1 pg/mL, p = 0.03), with a larger increase in *M. pneumoniae*-specific antibodies (+30 ± 15 vs. +5 ± 12 AU/mL, p = 0.001). Antibiotic use (6.5 ± 1.8 vs. 7.8 ± 2.0 days, p = 0.014) and total recovery time (8.2 ± 2.1 vs. 10.5 ± 2.5 days, p = 0.001) were shorter in the curcumin group. Nutritional indices showed improvement in hemoglobin (p = 0.01) and serum albumin (p = 0.02), while BMI showed a non-significant increase (p = 0.368). AE incidence was low and similar (6.3% vs. 8.8%, p = 0.55). In multivariable regression, curcumin remained independently associated with shorter recovery (β = −1.2, p = 0.001).

**Conclusion:**

Curcumin might be a safe and well-tolerated adjunct to standard antibiotic therapy in children with *M. pneumoniae* infections, potentially improving clinical outcomes, reducing inflammation, and supporting nutritional status.

## 1 Introduction

Curcumin, a bioactive compound derived from *Curcuma longa*, is well-recognized in traditional medicine for its potent antioxidant and anti-inflammatory properties (El-Saadony et al., 2022). Recent studies have further highlighted its potential roles in improving nutritional status and modulating immune responses (Allegra et al., 2022). *Mycoplasma pneumoniae*, a major cause of pediatric respiratory infections, poses significant challenges due to its atypical clinical presentation and resistance to standard antibiotic therapies (Ghoushi et al., 2024). Its capacity to evade host immunity and induce persistent inflammation underscores the need for adjunctive therapies that can enhance innate defense mechanisms. Nutritional interventions—particularly those involving bioactive compounds such as curcumin—are emerging as promising complements to conventional treatment strategies (Ghoushi et al., 2024).

Nutritional status plays a pivotal role in immune competence, with malnutrition or suboptimal nutrient intake impairing immune function and increasing susceptibility to infections (Kunnumakkara et al., 2023). Curcumin has been reported to activate immune cells, enhance antibody production, and regulate cytokine expression (Allegra et al., 2022). By addressing nutritional deficits and supporting immune function, curcumin may reduce the burden of chronic infections (Morales et al., 2023). In the context of *M. pneumoniae* infections, its anti-inflammatory effects are particularly relevant, given the protracted inflammatory responses typically observed in the respiratory tract (Ghoushi et al., 2024). Moreover, curcumin’s antioxidant activity may help mitigate oxidative stress, which is frequently associated with respiratory infections and delayed convalescence (Sathyab et al., 2022).

Given the limited therapeutic options for *M. pneumoniae* and the growing challenge of antibiotic resistance, the exploration of alternative or adjunctive therapies such as curcumin is increasingly warranted (Makuch et al., 2021). This study aims to investigate the mechanisms through which curcumin modulates immune and nutritional pathways in the context of *M. pneumoniae* infection and to evaluate its potential as a complementary treatment alongside standard antibiotic therapy.

## 2 Materials and methods

### 2.1 Study setting and participants

**FIGURE 1:**
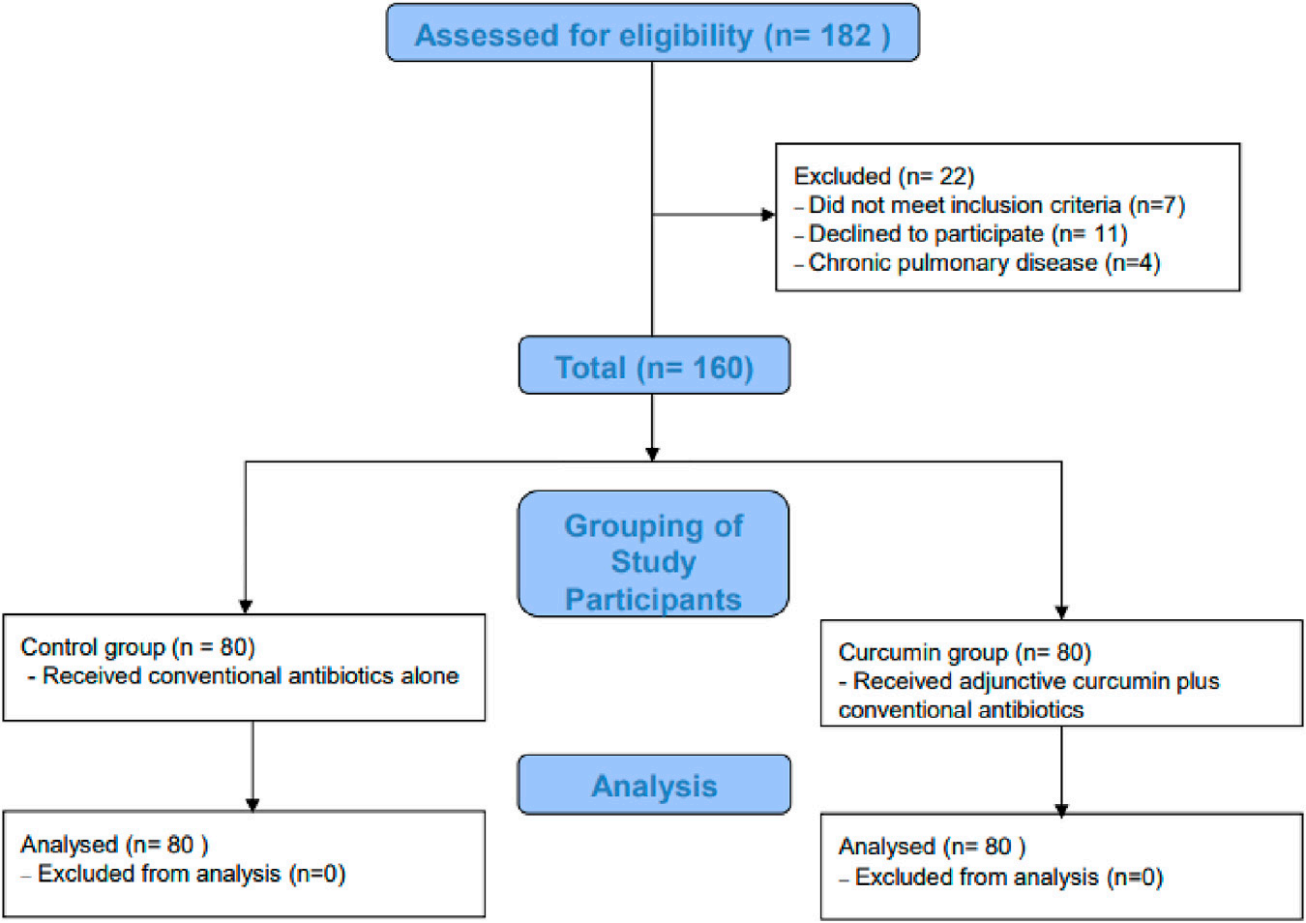
Flowchart of participant selection and grouping.

A retrospective observational study was performed in the Department of Pediatrics, Beijing Luhe Hospital, Capital Medical University. Medical records of children aged 1–12 years who were diagnosed with *M. pneumoniae* infection between September 2023 and May 2024 were reviewed. Inclusion criteria included children with clinical symptoms of respiratory infection and confirmed *M. pneumoniae* infection, based on serological (anti-*M. pneumoniae* Immunoglobulin M ≥ 1.1) or molecular evidence (Polymerase Chain Reaction Cycle Threshold ≤35 targeting the P1 adhesin gene) (Subspecialty Group of Respiratory et al., 2025). Exclusion criteria included chronic pulmonary diseases, immunosuppressive therapy, and a history of allergic reactions to curcumin or any components of the supplement. Informed consent was obtained from all subjects and their legal guardian(s). Our study’s methodology, design, and protocols underwent a rigorous review by our hospital’s ethics committee, aligning with the Declaration of Helsinki and relevant guidelines. All data was processed confidentially with personal identifiers removed to ensure privacy, upholding the highest ethical standards in medical research (Figure 1).

Infection severity (mild, moderate, severe) was classified based on a combination of clinical symptoms, radiological findings, and laboratory biomarkers, consistent with commonly applied pediatric guidelines (Subspecialty Group of Respiratory et al., 2025). Mild infection was defined as low-grade fever (<38.5 °C), mild respiratory symptoms (e.g., cough without dyspnea), oxygen saturation >95%, and limited radiographic changes without lobar consolidation. Moderate infection was defined as persistent fever (≥38.5 °C), moderate respiratory symptoms (e.g., frequent cough, mild to moderate dyspnea), radiographic evidence of extensive infiltrates, and CRP levels >10 mg/L but <40 mg/L. Severe infection was defined by any of the following: severe respiratory distress (tachypnea, SpO_2_ ≤92%), multilobar or segmental consolidation, CRP ≥40 mg/L, or complications such as pleural effusion or respiratory failure. Final severity classification was made by the treating physicians based on these criteria and documented clinical judgment.

### 2.2 Treatment classification

Subjects were classified into two cohorts according to supplemental therapy recorded in the medical chart: 1) the curcumin group, comprising patients who received adjunctive curcumin (standardized to 95% curcuminoids at 20 mg/kg/day mixed with food) in addition to conventional antibiotics; and 2) the control group, comprising patients managed with conventional antibiotics alone. Allocation to groups was determined by physician treatment decisions at the time of care; no randomization or blinding was performed. All patients received standard supportive measures, including oxygen therapy and antipyretics as indicated. All patients in both groups were treated with azithromycin as the first-line macrolide antibiotic, in accordance with institutional pediatric guidelines for *M. pneumoniae* infection. No alternative antibiotics were used in this cohort. The timing, dosage, and duration of curcumin administration were extracted from nursing and pharmacy records. Outcomes and adverse events were assessed through review of clinical notes, laboratory values, and imaging reports.

### 2.3 Data collection

Baseline data, including demographic characteristics (age, sex), nutritional status (height, weight, body mass index), and clinical parameters related to the severity of *M. pneumoniae* infection (fever, cough, dyspnea, and chest radiograph findings), were extracted from electronic medical records. Symptom progression, adverse effects, and supplement adherence were assessed based on documentation in nursing notes, caregiver reports recorded in the charts, and pharmacy dispensing records. Where available, adherence was estimated from recorded information on supplement use; patients with documented adherence below 80% were included in a sensitivity analysis to evaluate the robustness of the outcome measures.

### 2.4 Laboratory examinations

Laboratory data were obtained from hospital records, including results of blood samples collected at baseline (diagnosis) and after completion of treatment, as part of routine clinical care. Parameters included total white blood cell count, differential count, C-reactive protein (CRP), and specific antibodies against *M. pneumoniae*. In addition, levels of interleukin-6 (IL-6), tumor necrosis factor-alpha (TNF-α), and CRP were measured at two standardized timepoints: 1) baseline (at diagnosis, prior to treatment initiation) and 2) end of treatment (upon completion of the observation period). These measurements were performed using standard immunoassays in the hospital laboratory. No intermediate or serial measurements were available for this retrospective analysis. Specific antibodies against *M. pneumoniae* were quantified using a commercially available enzyme-linked immunosorbent assay (ELISA) kit. The assay measured anti-*M. pneumoniae* IgM antibodies and results were reported in arbitrary units per milliliter (AU/mL). IgM was selected as the primary serological marker for recent or active infection in pediatric patients. IgG antibodies were not routinely assessed in this study.

### 2.5 Outcome measures

The main outcome of interest was the duration of respiratory symptoms (fever and cough). Secondary outcomes included hospitalization rate, incidence of severe complications (e.g., pleural effusion, respiratory failure), and changes in immune function markers.

### 2.6 Statistical analysis

Data was analyzed using SPSS software (version 26.0; IBM Corp., Armonk, NY, United States). Descriptive statistics were presented as means ± standard deviations for continuous variables and as frequencies (percentages) for categorical variables. Between-group comparisons of continuous variables were performed using the independent samples t-test or the paired t-test for within-group comparisons. The chi-squared test or Fisher’s exact test was used for categorical variables, as appropriate. Multivariate linear regression analysis was conducted to identify independent predictors of recovery time, adjusting for potential confounders such as age, baseline CRP level, and initial disease severity. Subgroup analyses were performed stratified by age group and infection severity to assess the consistency of curcumin’s therapeutic effect. All statistical tests were two-sided, and a p-value <0.05 was considered statistically significant.

## 3 Results

### 3.1 Baseline demographic and clinical characteristics

Baseline demographic and clinical characteristics were comparable between the curcumin and control groups, with no statistically significant differences in age, sex distribution, weight, height, BMI, or initial symptoms such as fever, cough, and wheezing (all p > 0.05; Table 1).

**TABLE 1:** Baseline demographic and clinical characteristics of participants.

Variable	Curcumin group (n = 80)	Control group (n = 80)	p-value
Age (years)			
Mean ± SD	6.5 ± 3.1	6.3 ± 3.0	0.76
Sex (M/F)	40/40	42/38	0.74
Weight (kg)			
Mean ± SD	21.4 ± 4.5	21.1 ± 4.7	0.62
Height (cm)			
Mean ± SD	115.2 ± 15.3	114.8 ± 15.1	0.85
BMI (kg/m^2^)			
Mean ± SD	16.1 ± 1.4	16.0 ± 1.3	0.88
Initial Symptoms			
Fever (%)	75 (93.75)	74 (92.5)	0.82
Cough (%)	80 (100)	79 (98.75)	0.49
Wheezing (%)	30 (37.5)	28 (35)	0.70

At the end of the observation period, the curcumin group exhibited significantly shorter durations of fever and cough, as well as lower rates of hospitalization (1.25% vs. 10.0%) and severe complications (2.5% vs. 12.5%), compared to the control group (all p < 0.05; Table 2).

**TABLE 2:** Clinical outcomes at the end of the observation period.

Outcome	Curcumin group (n = 80)	Control group (n = 80)	p-value
Duration of Fever (days)			
Mean ± SD	3.2 ± 1.1	4.5 ± 1.3	0.01*
Duration of Cough (days)			
Mean ± SD	5.4 ± 2.0	7.1 ± 2.5	0.02*
Incidence of Severe Complications (%)	2 (2.5)	10 (12.5)	0.03*
Hospitalizations (%)	1 (1.25)	8 (10)	0.02*

### 3.2 Impact of curcumin on immune function markers

From baseline to the end of the observation period, as recorded in hospital records, the curcumin group showed a slight decrease in white blood cell count (from 6.5 ± 1.2 to 6.3 ± 1.1 ×10^9^/L), whereas no statistically significant change was observed in the control group (from 6.7 ± 1.3 to 6.9 ± 1.4 ×10^9^/L; p = 0.351). CRP levels decreased significantly in the curcumin group (from 20.2 ± 5.5 to 10.6 ± 4.2 mg/L), compared to a modest reduction in the control group (from 20.0 ± 5.7 to 18.2 ± 5.3 mg/L; p = 0.011). Specific antibody levels against *M. pneumoniae* increased from 50 ± 10 to 80 ± 15 AU/mL in the curcumin group, and from 50 ± 10 to 55 ± 12 AU/mL in the control group (p = 0.001; Table 3). The incidence of adverse effects, including gastrointestinal discomfort, allergic reactions, headaches, and rashes—was low and comparable between groups (p > 0.05; Supplementary Table S1).

**TABLE 3:** Changes in immune function markers from baseline to the end of the observation period.

Marker	Baseline (curcumin)	End of study (curcumin)	Baseline (control)	End of study (control)	p-value
White Blood Cells (x10^9^/L)	6.5 ± 1.2	6.3 ± 1.1	6.7 ± 1.3	6.9 ± 1.4	0.351
CRP (mg/L)	20.2 ± 5.5	10.6 ± 4.2	20.0 ± 5.7	18.2 ± 5.3	0.011*
Specific Antibodies (AU/mL)	50 ± 10	80 ± 15	50 ± 10	55 ± 12	0.001*

### 3.3 Safety and tolerability of curcumin

At the end of the observation period, patients in the curcumin group exhibited a modest but statistically significant improvement in nutritional indicators. BMI increased slightly from 16.1 ± 1.4 to 16.3 ± 1.4 kg/m^2^ (p = 0.368), while no significant change was detected in the control group. Hemoglobin levels rose from 11.2 ± 1.5 to 12.1 ± 1.3 g/dL in the curcumin group, compared to a marginal increase from 11.3 ± 1.4 to 11.4 ± 1.3 g/dL in the control group (p = 0.010). Likewise, serum albumin levels improved from 3.9 ± 0.5 to 4.2 ± 0.4 g/dL in the curcumin group, while remaining unchanged in the control group (p = 0.020; Table 4).

**TABLE 4:** Changes in nutritional status from baseline to the end of the observation period.

Nutritional parameter	Baseline (curcumin)	End of study (curcumin)	Baseline (control)	End of study (control)	p-value
BMI (kg/m^2^)	16.1 ± 1.4	16.3 ± 1.4	16.0 ± 1.3	16.0 ± 1.3	0.368
Hemoglobin (g/dL)	11.2 ± 1.5	12.1 ± 1.3	11.3 ± 1.4	11.4 ± 1.3	0.01*
Serum Albumin (g/dL)	3.9 ± 0.5	4.2 ± 0.4	3.9 ± 0.5	3.9 ± 0.5	0.02*

### 3.4 Effect of curcumin on inflammatory markers and recovery time

At the end of the study, the curcumin group showed a reduction in IL-6 levels from 45.2 ± 8.3 to 30.1 ± 6.5 pg/mL, while the control group showed a smaller decrease from 45.0 ± 8.5 to 42.5 ± 8.0 pg/mL (p = 0.010). TNF-α levels decreased from 35.0 ± 7.0 to 25.4 ± 6.0 pg/mL in the curcumin group and from 34.9 ± 7.1 to 33.2 ± 7.0 pg/mL in the control group (p = 0.030; Table 5).

**TABLE 5:** Inflammatory markers at baseline and at the end of the observation period.

Inflammatory marker	Baseline (curcumin)	End of study (curcumin)	Baseline (control)	End of study (control)	p-value
Interleukin-6 (IL-6) (pg/mL)	45.2 ± 8.3	30.1 ± 6.5	45.0 ± 8.5	42.5 ± 8.0	0.01∗
Tumor necrosis factor-alpha (TNF-α) (pg/mL)	35.0 ± 7.0	25.4 ± 6.0	34.9 ± 7.1	33.2 ± 7.0	0.03∗

The duration of antibiotic treatment was shorter in the curcumin group (6.5 ± 1.8 days) compared to the control group (7.8 ± 2.0 days; p = 0.014). Total recovery time was also reduced in the curcumin group (8.2 ± 2.1 days) *versus* the control group (10.5 ± 2.5 days; p = 0.001; Table 6).

**TABLE 6:** Antibiotic use and recovery time.

Measure	Curcumin group	Control group	p-value
Antibiotic Duration (days)	6.5 ± 1.8	7.8 ± 2.0	0.014∗
Total Recovery Time (days)	8.2 ± 2.1	10.5 ± 2.5	0.001∗

### 3.5 Subgroup and multivariate regression analysis of Curcumin’s effectiveness on recovery time

Multivariate regression analysis confirmed that curcumin supplementation was independently associated with a significantly shorter recovery time (β = −1.2, 95% CI: 1.8 to −0.6, p = 0.001), after adjusting for age, baseline CRP, and initial severity (Table 7). Notably, increased baseline severity (β = 1.3, p = 0.002), higher CRP levels (β = 0.05, p = 0.015), and older age (β = 0.1, p = 0.032) were independently associated with prolonged recovery.

**TABLE 7:** Multivariate analysis of factors associated with recovery time.

Factor	Coefficient	Standard error	p-value	95% confidence interval
Treatment Group	−1.2	0.3	0.001*	−1.8 to −0.6
Age	0.1	0.05	0.032*	0.01 to 0.2
Initial Severity	1.3	0.4	0.002*	0.5 to 2.1
Baseline CRP	0.05	0.02	0.015*	0.01 to 0.09

Subgroup analysis demonstrated a consistent benefit of curcumin across all age groups (1–3, 4–7, and 8–12 years) and infection severity levels (mild, moderate, and severe). In each subgroup, the curcumin group exhibited shorter mean fever durations compared to the control group. The most pronounced effect was observed in children aged 8–12 years with severe infection, where the mean fever duration was significantly shorter in the curcumin group (4.0 days) than in the control group (5.8 days; p < 0.01).

### 3.6 Durability of Curcumin’s therapeutic effects across demographic groups

Across all age groups (1–3, 4–7, and 8–12 years) and levels of initial severity (mild, moderate, severe), the curcumin group was observed to have shorter mean symptom durations, lower hospitalization rates, and reduced incidence of severe complications compared to the control group. Reductions in CRP levels were recorded in all curcumin subgroups, whereas minimal or no reductions were recorded in the corresponding control subgroups (Table 8).

**TABLE 8:** Detailed analysis of clinical outcomes by age group, treatment type, and initial severity.

Age group	Treatment	Initial Severity	Mean duration of symptoms (days)	Hospitalization Rate(%)	Sever complications Rate(%)	Change in CRP levels (mg/L)	p-value
1–3 Years	Curcumin	Mild	2.7	0.0	0.0	−10.2	0.01*
	Curcumin	Moderate	3.5	1.5	1.5	−9.2	0.05
	Curcumin	Severe	4.8	3.0	5.0	−8.7	0.04*
	Control	Mild	4.1	1.0	0.0	−0.3	0.86
	Control	Moderate	5.3	2.5	3.0	−0.1	0.92
	Control	Severe	7.0	8.5	10.0	0.5	0.88
4–7 Years	Curcumin	Mild	2.9	0.0	0.0	−12.0	0.01*
	Curcumin	Moderate	3.9	0.5	0.5	−11.4	0.05
	Curcumin	Severe	5.2	2.5	4.0	−10.0	0.02*
	Control	Mild	4.4	0.5	0.0	−1.2	0.84
	Control	Moderate	6.0	3.0	3.5	−0.8	0.90
	Control	Severe	8.1	10.0	11.5	1.0	0.86
8–12 Years	Curcumin	Mild	3.0	0.0	0.0	−13.5	0.03*
	Curcumin	Moderate	4.0	1.0	1.5	−12.9	0.04*
	Curcumin	Severe	6.0	4.0	6.0	−11.5	0.04*
	Control	Mild	4.6	1.0	0.5	−2.1	0.83
	Control	Moderate	6.5	5.0	5.5	−1.5	0.89
	Control	Severe	9.2	13.0	14.0	2.0	0.87

## 4 Discussion

This study suggests that curcumin, when used as an adjunct to standard antibiotic therapy, was associated with improvements in clinical, immunological, and nutritional outcomes in children with *M. pneumoniae* infection. Patients receiving curcumin had shorter recorded durations of fever and cough, fewer hospitalizations, and a lower incidence of severe complications. Reductions in inflammatory markers (CRP, IL-6, TNF-α) and increases in pathogen-specific antibody levels were observed, which may reflect enhanced immune responses (Munteanu and Schwartz, 2022). Moreover, curcumin supplementation was associated with improvements in hemoglobin and serum albumin levels, despite the relatively short supplementation period. These associations were consistent across age groups and severity levels and were supported by multivariate analysis of retrospective data, in which curcumin supplementation was an independent predictor of shorter recovery time. Collectively, these findings support the potential role of curcumin as a safe adjunctive therapy in the management of pediatric respiratory infections caused by *M. pneumoniae*, while emphasizing the need for confirmation in prospective randomized controlled trials (Nosrati-Oskouie et al., 2022).

Compared to the control group, the curcumin-supplemented group exhibited a significantly shorter duration of fever and respiratory symptoms, along with lower rates of hospitalization and severe complications. These clinical benefits align with curcumin’s well-documented anti-inflammatory and immunomodulatory properties, which may help regulate host immune responses during infection (Peng et al., 2021; Uchio et al., 2021). The observed post-treatment reductions in TNF-α, IL-6, and CRP levels likely reflect curcumin’s capacity to downregulate pro-inflammatory cytokine production, as reported in prior studies. Additionally, the shorter duration of antibiotic use in the curcumin group may indicate enhanced endogenous immune defense—a critical factor given rising concerns about antimicrobial resistance (Vahedian-Azimi et al., 2022). Our findings are consistent with previous research, including the work of Ghoushi et al. (Ghoushi et al., 2024), which demonstrated that curcumin could modulate a broad spectrum of immunological and inflammatory biomarkers. Collectively, these observations suggest that curcumin may improve clinical outcomes in pediatric *M. pneumoniae* infection by attenuating excessive inflammation and supporting immune resolution (Wang et al., 2024).

The observed reductions in TNF-α and IL-6 levels in the curcumin group are likely to reflect its established anti-inflammatory mechanisms, including inhibition of nuclear factor kappa-light-chain-enhancer of activated B cells (NF-κB) and modulation of the extracellular signal-regulated kinase 1/2 (ERK1/2) and c-Jun N-terminal kinase (JNK) signaling pathways (Iglesias et al., 2022). Curcumin has also been shown to promote IL-6R shedding via disintegrin and metalloproteinase domain-containing protein 10 (ADAM10), potentially dampening downstream IL-6 signaling (Murai et al., 2025). These mechanistic insights align with our findings of significantly decreased CRP, TNF-α, and IL-6 levels following curcumin supplementation. Additionally, curcumin enhanced humoral immunity, as evidenced by increased M. pneumoniae-specific IgM levels (Rytter et al., 2014). Taken together, these results suggest that curcumin exerts dual immunomodulatory effects, suppressing excessive inflammatory responses while promoting antigen-specific antibody production, thereby contributing to clinical improvement and reduced complication rates in pediatric *M. pneumoniae* infections (Yang, 2019). The significant increase in *M. pneumoniae*–specific antibodies observed in the curcumin group suggests a potential immunomodulatory effect, enhancing the host’s ability to mount a more effective pathogen-specific immune response. This finding aligns with the results of Afolayan et al. (Afolayan et al., 2018), who reported that curcumin can augment immune defense mechanisms in infected hosts by modulating antibody production. Beyond infection control, curcumin supplementation was associated with improvements in nutritional parameters, including serum albumin, hemoglobin levels, and body mass index. These findings are noteworthy, as immune competence is closely linked to nutritional status, and malnutrition is a well-recognized risk factor for impaired immunity and increased infection susceptibility. Thus, curcumin may support host resilience not only by modulating immune responses but also by contributing to nutritional recovery (Xu et al., 2018). Thus, curcumin may enhance host resilience not only by modulating immune responses but also by supporting nutritional recovery.

Curcumin demonstrated a favorable safety profile, with no significant increase in adverse effects observed compared to the control group—an important consideration in pediatric populations where tolerability is paramount (Khalili and Nammi, 2022). This finding is consistent with prior studies confirming the safety of curcumin even at higher doses (Suresh et al., 2023). While the observed increase in BMI from 16.1 to 16.3 kg/m^2^ did not reach statistical significance (p = 0.368), the direction of change may still suggest a trend toward improved nutritional status. However, the absolute change was modest and may have limited clinical relevance over the short supplementation period. Nevertheless, the concurrent improvements in hemoglobin and serum albumin levels may indicate a broader trend toward enhanced nutritional status, warranting further evaluation in prospective, longer-term studies. Despite curcumin’s inherently low oral bioavailability due to poor absorption and rapid metabolism, the observed systemic effects—including reductions in TNF-α and IL-6—suggest potential gastrointestinal immune modulation or cumulative effects from repeated dosing. Although unmodified curcumin (standardized to 95% curcuminoids) was used without bioenhancers, prior studies indicate that even low systemic levels may exert biological activity via gut-mediated signaling pathways (Scazzocchio et al., 2020; Zhu and He, 2024). Future formulations with enhanced pharmacokinetics (e.g., nanoparticles, liposomes, or piperine co-administration) may further optimize clinical efficacy.

This study has several limitations. Conducted at a single center, it may limit the generalizability of findings to broader pediatric populations or different clinical settings. The non-randomized and non-blinded nature of the study introduces selection and observer biases, as treatment allocation was based on the physician’s clinical decisions, potentially influenced by patient characteristics and illness severity. Additionally, the short supplementation and observation periods restrict our ability to evaluate the long-term effects of curcumin, including recurrence rates and delayed adverse events. While improvements in BMI, hemoglobin, and serum albumin were statistically significant, their clinical relevance is uncertain due to the modest changes observed. Curcumin’s immunomodulatory effects were inferred from cytokine and antibody level changes, but direct molecular or cellular mechanisms were not assessed. Future research should address these limitations by conducting multi-center, prospective, randomized controlled trials (RCTs) with larger sample sizes to minimize biases and confounders. These studies would provide stronger evidence for curcumin’s efficacy and safety in pediatric *M. pneumoniae* infections, with longer follow-up periods to assess sustained efficacy and potential delayed effects. Mechanistic studies using genomic, proteomic, and metabolomic approaches are essential to better understand curcumin’s molecular pathways. Additionally, evaluating curcumin in combination with other nutraceuticals could identify synergistic effects, enhancing clinical outcomes.

## 5 Conclusion

This study suggests that curcumin might serve as a promising adjunctive therapy for pediatric *M. pneumoniae* infection, potentially reducing symptom duration and the incidence of severe complications. The observed associations with improvements in clinical, immunological, and nutritional parameters could reflect its anti-inflammatory and immunomodulatory properties. Moreover, its favorable safety profile indicates that curcumin could be a well-tolerated and potentially beneficial option in the management of pediatric infectious diseases, warranting confirmation in prospective randomized trials.

## Data Availability

The raw data supporting the conclusions of this article will be made available by the authors, without undue reservation.
