# Supplementary material for: The role of curcumin in modulating nutritional status and susceptibility to Mycoplasma pneumoniae infection in children
Source: Front Pharmacol. 2025 Sep 11;16:1651875. doi: 10.3389/fphar.2025.1651875 (PMC12462405; doi:10.3389/fphar.2025.1651875)
Supplement: Supplementary file 1 [file Table1.docx]

**Supplementary Table S1.** Summary of adverse events observed in the curcumin and control groups during the study period

| **Adverse Event** | **Curcumin Group (n=80)** | **Control Group (n=80)** | **p-value** |
| --- | --- | --- | --- |
| Gastrointestinal discomfort | 2 (2.5%) | 3 (3.8%) | 0.65 |
| Allergic reaction (mild) | 1 (1.3%) | 1 (1.3%) | 1.00 |
| Headache | 1 (1.3%) | 2 (2.5%) | 0.56 |
| Rash | 1 (1.3%) | 1 (1.3%) | 1.00 |
| **Total incidence** | 5 (6.3%) | 7 (8.8%) | 0.55 |
